# Chronic Nerve Growth Factor Exposure Increases Apoptosis in a Model of In Vitro Induced Conjunctival Myofibroblasts

**DOI:** 10.1371/journal.pone.0047316

**Published:** 2012-10-10

**Authors:** Alessandra Micera, Ilaria Puxeddu, Bijorn Omar Balzamino, Stefano Bonini, Francesca Levi-Schaffer

**Affiliations:** 1 IRCCS - G.B. Bietti Foundation, Rome, Italy; 2 Department of Pharmacology and Experimental Therapeutics, School of Pharmacy, Institute for Drug Research, Faculty of Medicine, The Hebrew University of Jerusalem, Jerusalem, Israel; 3 Department of Ophthalmology, University Campus Bio-Medico, Rome, Italy; University of Florida, United States of America

## Abstract

In the conjunctiva, repeated or prolonged exposure to injury leads to tissue remodeling and fibrosis associated with dryness, lost of corneal transparency and defect of ocular function. At the site of injury, fibroblasts (FB) migrate and differentiate into myofibroblasts (myoFB), contributing to the healing process together with other cell types, cytokines and growth factors. While the physiological deletion of MyoFB is necessary to successfully end the healing process, myoFB prolonged survival characterizes the pathological process of fibrosis. The reason for myoFB persistence is poorly understood. Nerve Growth Factor (NGF), often increased in inflamed stromal conjunctiva, may represent an important molecule both in many inflammatory processes characterized by tissue remodeling and in promoting wound-healing and well-balanced repair in humans. NGF effects are mediated by the specific expression of the NGF neurotrophic tyrosine kinase receptor type 1 (trkA^NGFR^) and/or the pan-neurotrophin glycoprotein receptor (p75^NTR^). Therefore, a conjunctival myoFB model (TGFβ1-induced myoFB) was developed and characterized for cell viability/proliferation as well as αSMA, p75^NTR^ and trkA^NGFR^ expression. MyoFB were exposed to acute and chronic NGF treatment and examined for their p75^NTR^/trkA^NGFR^, αSMA/TGFβ1 expression, and apoptosis. Both NGF treatments significantly increased the expression of p75^NTR^, associated with a deregulation of both αSMA/TGFβ1 genes. Acute and chronic NGF exposures induced apoptosis in p75^NTR^ expressing myoFB, an effect counteracted by the specific trkA^NGFR^ and/or p75^NTR^ inhibitors. Focused single p75^NTR^ and double trkA^NGFR^/p75^NTR^ knocking-down experiments highlighted the role of p75^NTR^ in NGF-induced apoptosis. Our current data indicate that NGF is able to trigger *in vitro* myoFB apoptosis, mainly via p75^NTR^. The trkA^NGFR^/p75^NTR^ ratio in favor of p75^NTR^ characterizes this process. Due to the lack of effective pharmacological agents for balanced tissue repairs, these new findings suggest that NGF might be a suitable therapeutic tool in conditions with impaired tissue healing.

## Introduction

Acute and chronic inflammation of the conjunctiva causes the alteration of local architecture with tissue remodeling and fibrosis associated with ocular dryness and corneal complications leading to visual function impairment [1]. Traumatic, chemical, inflammatory or infectious insults, as well as surgical scarring, all represent promoting-causes of ocular fibrosis [2]. Pharmacological management and/or surgical strategies are required to restore healthy conjunctival structure, function and ocular transparency of compromised cornea. Conjunctival Fibroblasts (FB) and their differentiated α Smooth Muscle Actin (αSMA) expressing myofibroblasts (myoFB) play a pivotal role during repair/remodeling processes as targets, effectors and modulators of the process [3]. MyoFB appearance at the early phases and disappearance at the late phases of tissue repair are necessary steps to gain proper tissue healing [4]. MyoFB persistence seems to be one of the causes of fibrosis and may be due to an “acquired resistance to cell apoptosis” and/or to a microenvironment suitable for FB prolonged activity and survival [5]. Apoptosis of myoFB has been documented *in vivo*, representing a physiological process [3]. More than a few soluble factors (TGFβ1, IL6, IL4, IL13, etc.) have been reported to promote or counteract (IL1β, IL7) FB/myoFB physiological balance [6].

Several findings suggest the contribution of either endogenously produced or topically applied Nerve Growth Factor (NGF) in healing processes, wound-narrowing, tissue remodeling and fibrosis processes [6]–[9]. Tears, as well as inflamed conjunctiva, are characterized by increased TGFβ1 and NGF, a consequence of the inflamed microenvironment [10], [11]. NGF is a pleiotrophic factor that promotes cell growth, differentiation, survival and death among different tissues [7], [8], [11]. NGF activities appear to be mediated by two different receptors: the specific NGF neurotrophic tyrosine kinase receptor type 1 (trkA^NGFR^) of 140 kDa and the pan-neurotrophin low affinity glycoprotein receptor (p75^NTR^) of 75 kDa, a typical death receptor belonging to the tumor necrosis receptor superfamily [8], [12]. Our group previously reported that human conjunctival FB express constitutive trkA^NGFR^ and differentiate into αSMA and p75^NTR^ bearing myoFB upon NGF stimulation, suggesting NGF contribution at wound-narrowing and during healing of ulcers [13], [14]. Even though NGF withdrawal is largely considered the cause of NGF-associated cell death, several findings point to NGF as both a pro- and anti-apoptotic factor. A specific cell-surface trkA^NGFR^/p75^NTR^ ratio seems to be directly responsible for either proliferative and/or survival effects (trkA^NGFR^) or apoptotic response (p75^NTR^), with p75^NTR^ acting alone or in combination to modulate trkA^NGFR^ trafficking and/or signaling [12], [15], [16]. NGF-induced myoFB conversion was associated with the selective expression of p75^NTR^ [12].

To test whether NGF exposure might modulate myoFB behavior, we induced in vitro the myoFB phenotype via conjunctival FB exposure to TGFβ1. Here we describe evidence that conjunctival TGFβ1-induced myoFB express p75^NTR^ and are more sensitive to apoptosis after NGF treatment.

## Results

### Acute and Chronic NGF Exposure Increases p75^NTR^ Expression but does not Influence trkA^NGFR^ Expression

**Figure 1:**
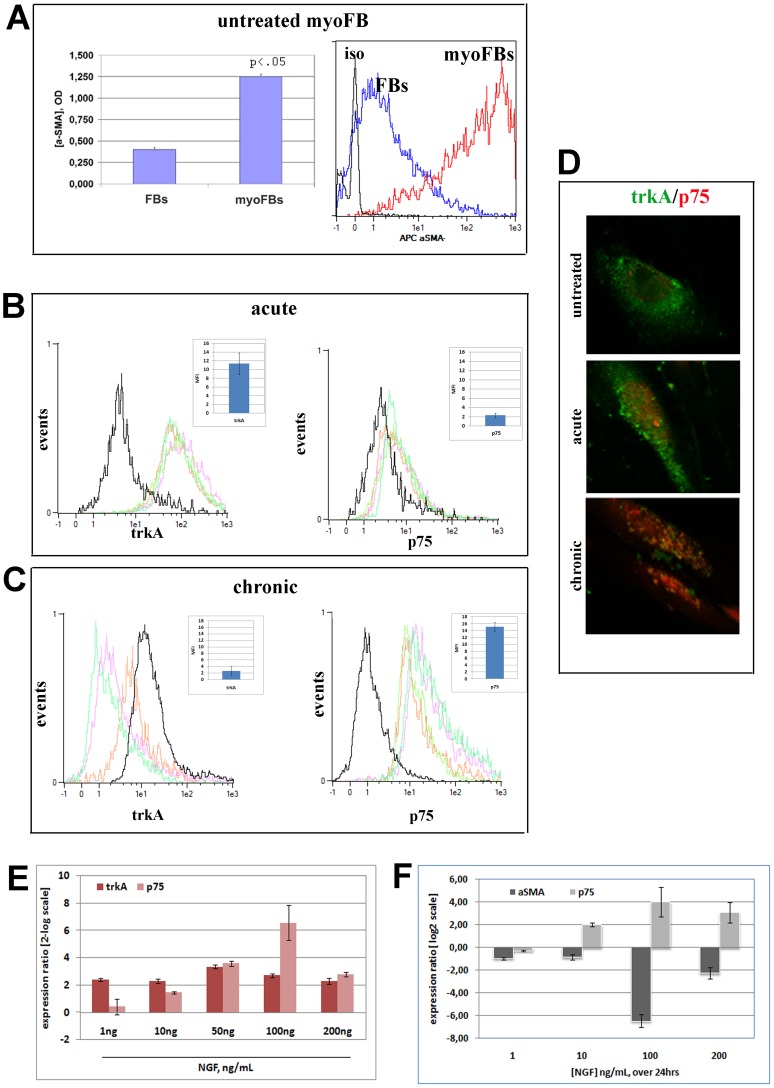
Characterization of TGFβ1-induced myoFB. Confluent myoFB were exposed to NGF at the indicated doses/times and evaluated for biochemical and molecular changes. (A) αSMA protein expression in both myoFB and FB, as detected by cell surface ELISA (optic density) and flow cytometry (fluorescence intensity). (B–C) Fluorescent histograms showing a different pattern of trkA^NGFR^ and p75^NTR^ expression in acute and chronic 100 ng/mL NGF exposed myoFB, as compared to untreated myoFB (black line). Mean Fluorescent Intensity (MFI) plots are shown inside fluorescent histograms (iso stands for isotype-matched control FI; 7.05 for trkA^NGFR^ and 8.39 for p75^NTR^). (D) Morphological distribution of trkA^NGFR^/p75^NTR^ in untreated, acute and chronic 100 ng/mL NGF treated myoFB. (E–F) Target gene expression specific for trkA^NGFR^/p75^NTR^ after acute and for αSMA/p75^NTR^ after chronic exposure to increasing NGF (p<.05).

Conjunctival FB were exposed to 2 ng/mL TGFβ1 for 3 days to develop the myoFB phenotype, according to previous studies [17]. Induced myoFB were re-plated at high density (to retain myoFB phenotype) and once at confluence were exposed to single (mimicking acute treatment) or three repeated NGF doses every two days (mimicking chronic treatment), with sampling at the specified times from the last stimulation. TGFβ1-induced myoFB (herein referred to as myoFB) showed a significant high expression of αSMA protein, as detected by confocal microscopy, cell surface ELISA and flow cytometry (Figure 1A). These myoFB expressed both trkA^NGFR^ and p75^NTR^, as shown by flow cytometry (Figure 1B-C; black lines).

When the myoFB were exposed to NGF (100 ng/mL) and harvested 24 hrs from the last stimulation, an increase of p75^NTR^ protein (Mean Fluorescent Intensity, MFI) was detected in both acute (ΔMFI = 90.25, range 57.00 to 173.20; Figure 1B) and more significant chronic (ΔMFI = 143.50, range 120.30 to 184.00; Figure 1C) treatments, as compared to untreated ones (ΔMFI = 58.00, range 56.00 to 60.80; p<.05; black lines). The morphological distribution of both trkA^NGFR^ and p75^NTR^ in untreated, acute and chronic NGF-treated myoFB (48 hrs from last stimulus) is shown in Figure 1D. The molecular analysis carried out on myoFB exposed to increasing NGF (0–200 ng/mL) showed the upregulation of p75^NTR^ target gene as soon as 5 hrs from stimulation, while unchanged values characterized trkA^NGFR^ expression (Figure 1E). p75^NTR^ expression showed a linear increase between 1 and 100 ng/mL (R^2^ = 0.996, p75^NTR^ ratios vs. NGF doses). The maximum p75^NTR^ increase was detected at 100 ng/mL NGF (trkA^NGFR^: 2.71±0.12 and p75^NTR^ 6.56±1.28 [2-log scale]), as compared to untreated myoFB (trkA^NGFR^: 1.10±0.05 and p75^NTR^: 1.80±0.71 [2-log scale] untreated myoFB vs. untreated FB; p<.05). The concentration of 100 ng/mL NGF resulted in a trkA^NGFR^/p75^NTR^ rate shift in favor of p75^NTR^ (trkA^NGFR^/p75^NTR^ ratio: 0.41; p<.05), in comparison to those observed in untreated myoFB (trkA^NGFR^/p75^NTR^ ratio of 2.83 in favor of trkA^NGFR^; p<.05). A negative correlation was found between trkA^NGFR^/p75^NTR^ at acute and chronic treatments (Rho = -0.782; p = .01; Spearman’s rank test). At the same time, a significant TGFβ1 down-regulating expression was detected at 10 and 100 ng/mL NGF (respectively −7.09±0.012 and −7.59±0.02 [2-log scale]). Interestingly TGFβ1, used as internal control, did not modulate its own TGFβ1 gene expression at 10 ng/mL (0.03±0.01 [2-log scale]).

p75^NTR^ over-expression was associated with αSMA protein (data not shown) and mRNA down-regulation, especially after chronic NGF exposure (Figure 1F). A significant 6.8-fold decrease in αSMA expression was detected after 100 ng/mL chronic NGF exposure while a slight decrease was observed at all the other concentrations (1 ng/mL NGF: 1.30-fold decrease, 10 ng/mL NGF: 1.10-fold decrease, 200 ng/mL NGF: 2.00-fold decrease; p<.05). αSMA-expressing myoFB showed p75^NTR^ mainly localized at the nuclear membrane or along the cytoskeleton (data not shown). A direct correlation was found between αSMA and p75^NTR^ upon chronic NGF exposure (Rho = 0.702; p = .002, Spearman’s rank test), implying that the decrease of both markers occurred alongside chronic NGF exposure.

### Acute and Chronic NGF Exposure Triggers TUNEL and AnnexinV Positive Cells

**Figure 2:**
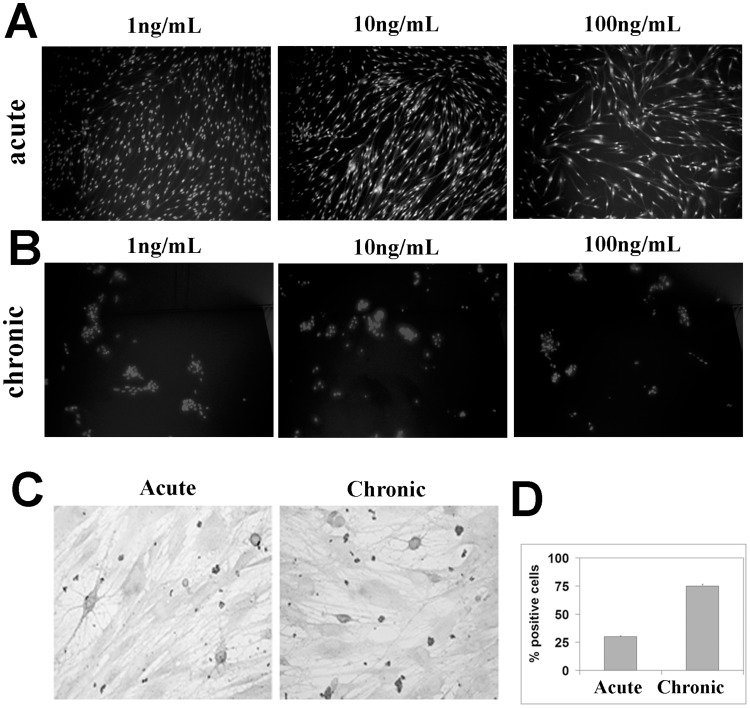
Micrographs of acute and chronic NGF exposed myoFB. Cells were exposed to different concentrations of NGF and stained with fluorescent DAPI or colorimetric TUNEL. (A-B) An increase of fluorescent cells showing nuclear condensation, picnotic nuclei and perimembrane vescicles is noticeable after acute and chronic NGF treatments. (C) TUNEL images from acute and chronic NGF exposed monolayers. (D) Histogram showing the % of TUNEL-positive NGF treated myoFB, over untreated ones (p<.05).

Since a decrease in the number of cells and viability were detected after acute and chronic NGF exposure, NGF exposed monolayers were stained with HO342 and/or DAPI and observed by fluorescence microscopy. Cells with clear signs of nuclear fragmentation were visible as soon as 5 hrs after acute NGF exposure. As shown, acute (Figure 2A **)** and chronic (Figure 2B) NGF exposure increased the number of rounded, crescent and condensed cells as well as the blabbed chromatine, indicating that myoFB were undergoing apoptosis in a time and a dose dependent manner.

TUNEL-reactivity confirmed the presence of *in situ* oligonucleosomal fragmentation. TUNEL-positive cells were quantified after acute and chronic NGF treatment, by counting in a blind fashion those myoFB showing brown-picnotic nuclei in different random fields (Figure 2C). Data were statistically compared to those of untreated myoFB (Figure 2D). Since some apoptotic nuclei were devoid of visible cytoplasm, the number of apoptotic cells was likely to be underestimated (Figure 2D vs. Figure 2A,B). Few TUNEL-positive myoFB were also detected in untreated cultures of myoFB (data not shown).

**Figure 3:**
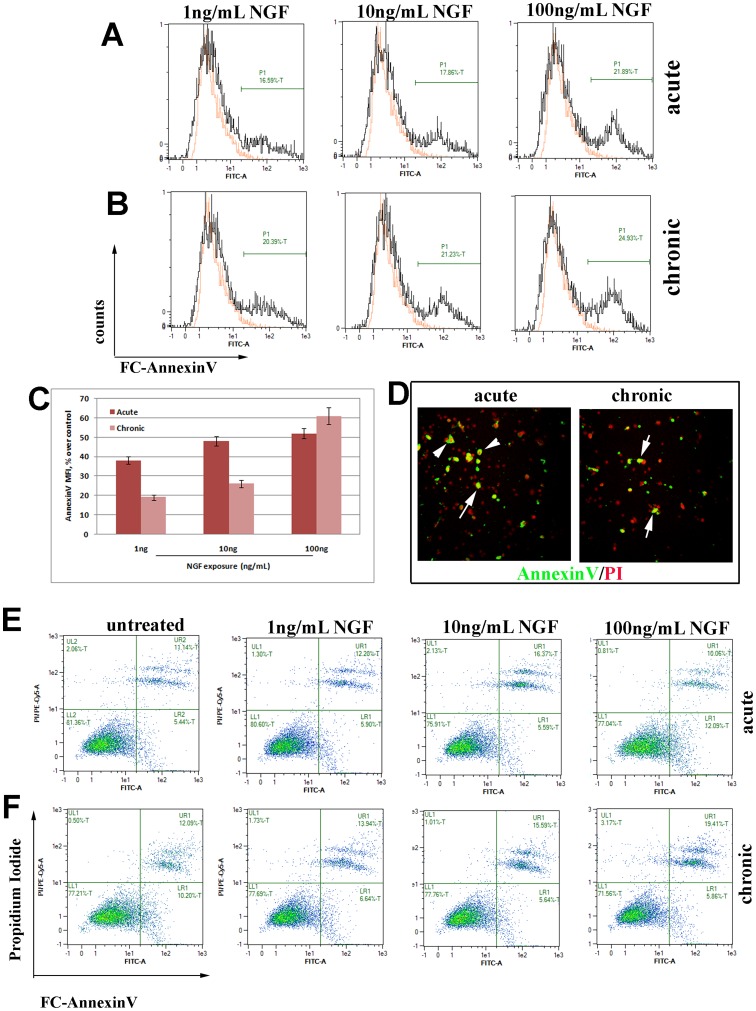
AnnexinV/PI analysis. (A–B) Fluorescent histograms of unsorted (total) cells exposed to acute and chronic NGF doses up to 24 hrs, showing an increased % of AnnexinV positive cells (isotype-matched control FI = 7.65). (C) AnnexinV specific Mean Fluorescent Intensity (MFI) for both acute and chronic, as a function of NGF treatments (% over control untreated myoFB, p<.05). (D) Representative pictures depicting monolayers exposed to acute or chronic 100 ng/mL NGF showing perinuclear/nuclear AnnexinV positivity alone (arrow-heads) or in combination with Propidium Iodide (PI; arrows) in stained cells. (E–F) Cytograms showing AnnexinV/PI plotter in cells exposed to acute and chronic exposure to NGF. Untreated myoFB were mainly AnnexinV/PI double-negative (LL, Lower Left quadrant), with a % range between 73 and 85; both acute and chronic NGF treatments resulted in increases of both AnnexinV^pos^/PI^neg^ (LR, Lower Right quadrant), indicating a proportion of cells undergoing apoptosis. Particularly, upon chronic NGF treatment, a population of cells also progressed to a later stage of apoptosis (AnnexinV^pos^/PI^pos^; UR, Upper Right quadrant).

Additional studies were performed by using AnnexinV/caspase staining. Outer membrane exposure of phosphatidylserine (PS) was detected by incubating unfixed cells with FC-conjugated AnnexinV. As shown, AnnexinV positivity increased in myoFB after acute (Figure 3A) and chronic (Figure 3B) NGF treatments. The intensity of FC-AnnexinV binding was quantified by flow cytometry (MFI), resulting in a significant increase of % MFI after acute NGF exposure and more consistent after chronic NGF exposure, as compared to untreated cells (Figure 3C). As shown by confocal microscope analysis (Figure 3D), AnnexinV was detected at the cell membrane of fixed myoFB counterstained with Propidium Iodide (PI). To better discriminate between early and late apoptotic versus necrotic cells, cytograms of FC-AnnexinV versus PI were analysed. As shown in Figure 3E–F, untreated myoFB were mainly AnnexinV ^negative/^PI ^negative^, indicating high viability and low apoptosis/necrosis. After acute treatment with increasing doses of NGF (Figure 3E), a significant number of AnnexinV ^positive/^PI ^negative^ cells (early stages of apoptosis) with a low proportion of AnnexinV ^positive/^PI ^positive^ (late stages of apoptosis) was found. This increase was associated with NGF treatment. Upon chronic exposure (Figure 3F), cells progressed to late stages of apoptosis, as detected by a significant increase of AnnexinV ^positive/^PI ^positive^ rate (Figure3C–D). The presence of AnnexinV ^positive/^PI ^negative^ cells in untreated myoFB, an indicator of early apoptotic myoFB, might be explained as a physiological event occurring in cultured myoFB.

### p75^NTR^-bearing MyoFB Undergo Apoptosis via Caspase3 Activation

**Figure 4:**
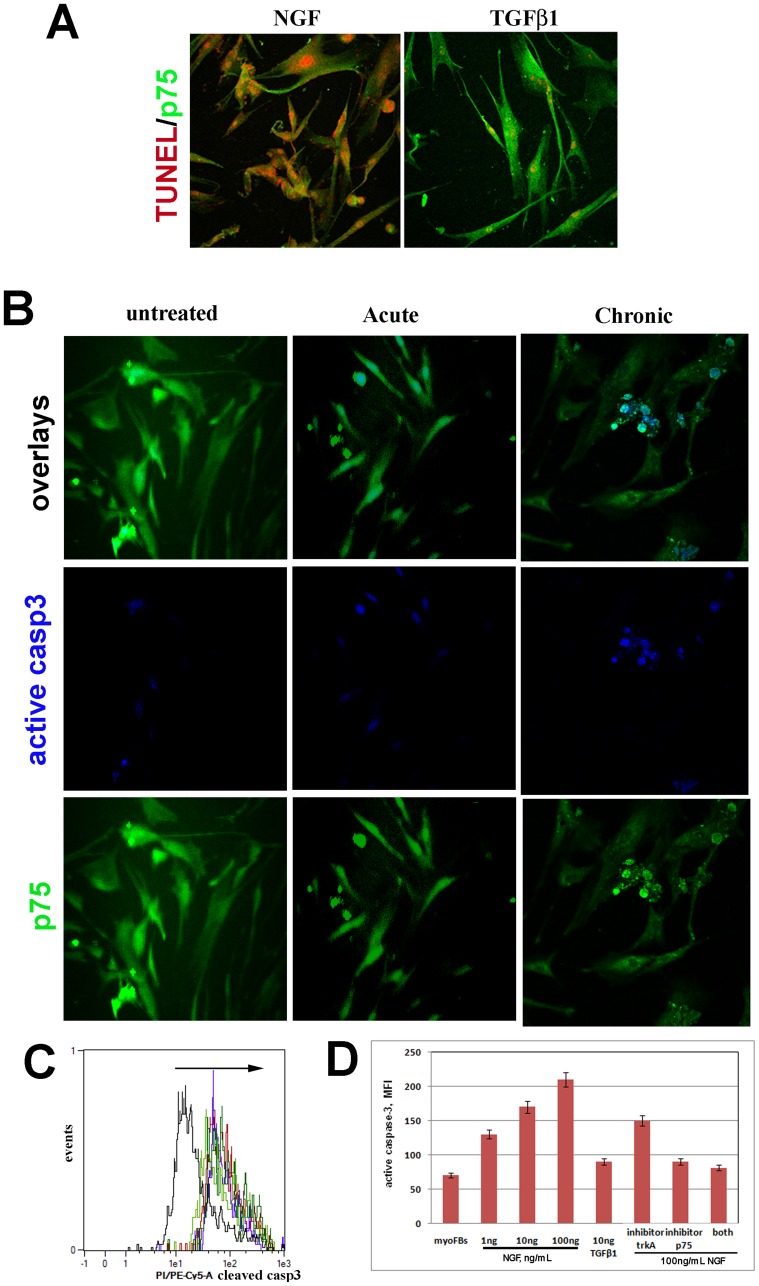
p75^NTR^-bearing apoptotic cells express caspase3. (A) Pictures depicting TUNEL reactivity in p75^NTR^ positive cells exposed to chronic 100 ng/mL NGF, as compared to TGFβ1 exposed cells. Changes in morphology, such as having rounded cytoplasm and markedly condensed chromatin/nuclear fragmentation, are clear visible in p75^NTR^-bearing myoFB. A few TUNEL-positive myoFB were also quantified in untreated cultures (not shown). (B) Overlays and single staining specific for p75^NTR^ and cleaved caspase3 are visible in both acute and chronic 100 ng/mL NGF exposed myoFB, as compared to untreated myoFB. (C–D) p75^NTR^ positive sorted cells analysed for active caspase3 expression and related statistical analysis of MFI. Note the specific reduction upon pretreated with trkA^NGFR^ and/or p75^NTR^ chemical inhibitors.

As observed by studies of double staining, p75^NTR^ expressing myoFB showed round condensed chromatin (picnotic) nuclei. This TUNEL/p75^NTR^ coexpression was particularly evident for 100 ng/mL NGF treated myoFB, in comparison to TGFβ1 treated ones (Figure 4A). By itself, TGFβ1 treatment did not significantly influence the number of apoptotic cells.

In these p75^NTR^ bearing cells, the contribution of caspase pathway was also investigated by using terminal effector caspase3 antibodies. Overlays and single staining specific for cleaved (active) caspase3 or p75^NTR^ expression are shown in untreated, acute and chronic NGF exposed myoFB (Figure 4B). The round morphology and the p75^NTR^/cleaved caspase3 coexpression are particularly visible in chronic NGF exposed myoFB with respect to untreated myoFB. Flow cytometry showed that cleaved caspase3 was increased in NGF exposed myoFB, in a dose-dependent fashion, with a maximum effect at 100 ng/mL NGF. A selected p75^NTR^-bearing population exposed to 100 ng/mL NGF for 12 hrs and showing an increase of cleaved caspase3 is plotted in Figure 4C.

Since myoFB express both trkA^NGFR^/p75^NTR^ on their surface, NGF-induced apoptosis was also investigated in the presence of trkA^NGFR^ and p75^NTR^ specific inhibitors, alone or in combination. As noted, active caspase3 signal was significantly reduced after preincubation with trkA^NGFR^ and/or p75^NTR^ chemical inhibitors (500 ng/mL; Figure 4D). This effect suggests that both low and high trkA^NGFR^/p75^NTR^ rates are involved in the apoptotic process. A low degree of apoptotic signal was detected in both untreated and TGFβ1-treated myoFB.

### p75^NTR^ Inhibits and trkA^NGFR^ Contributes to NGF-induced MyoFB Apoptosis

**Figure 5:**
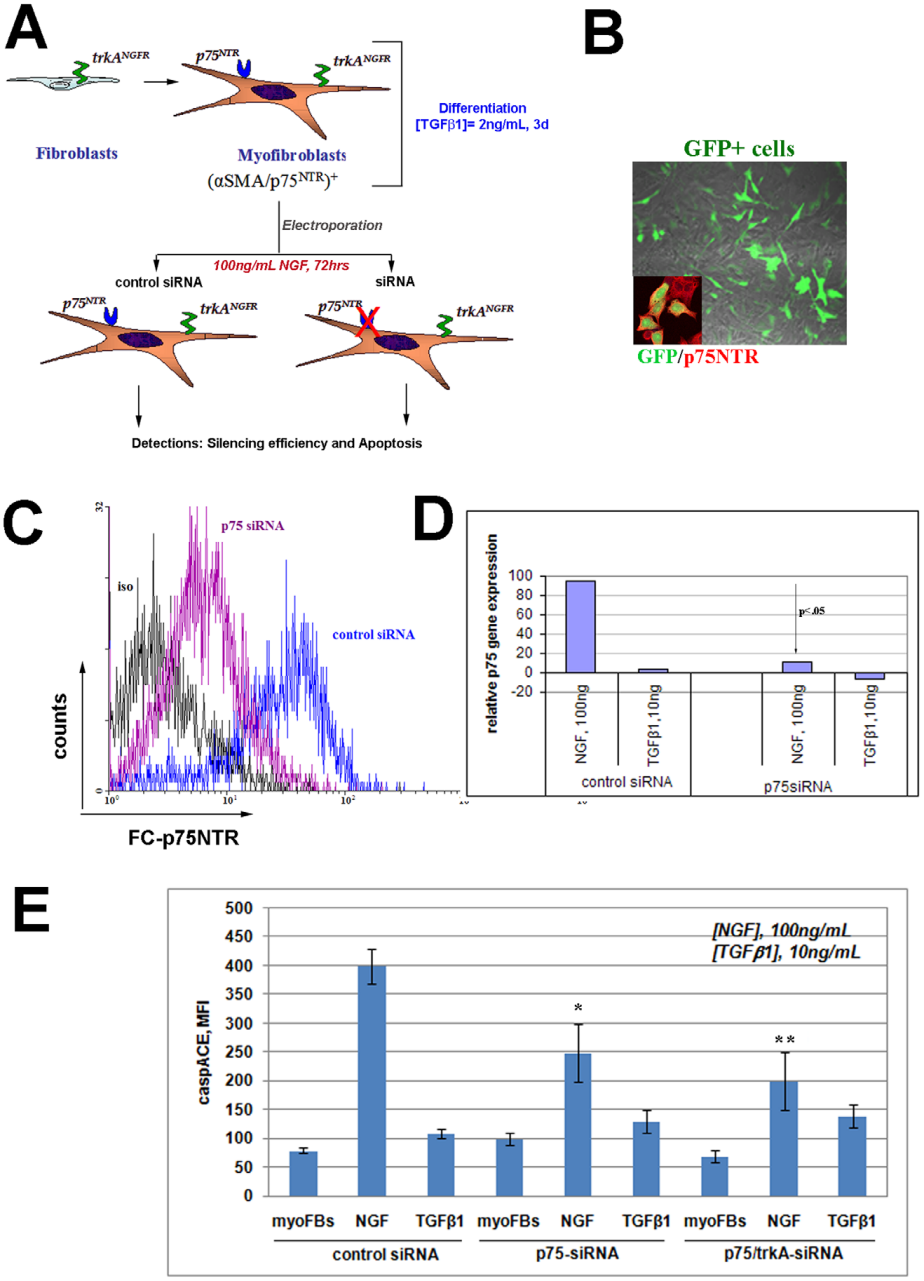
Receptor specific silencing decreases NGF induced apoptosis. (A) A flow chart schematizing the siRNA experiments. (B) Confocal images depicting GFP positive cells (image) and p75^NTR^-GFP double-positive cells (insert over the image), re-plated 5 hrs after electroporation. (C–D) Representative histogram for GFP specific staining, in comparison to control siRNA (control cells), showing a decreased p75^NTR^ positive expression and the related p75^NTR^ target gene deregulation. (E) Mean Fluorescence Intensity (MFI) specific for caspACE-VAD comparing 100 ng/mL NGF and 10 ng/mL TGFβ1 effects on control-siRNA, p75^NTR^ siRNA and double trkA^NGFR^/p75^NTR^ siRNA treated cells. A 37.5%-decrease (*) and a 50%-decrease (**) of apoptotic signal were detected respectively in p75^NTR^-siRNA and p75^NTR^/trkA^NGFR^-siRNA (p<.05).

To provide further information on p75^NTR^ and particularly trkA^NGFR^/p75^NTR^ contribution in NGF-mediated apoptosis, appropriate silencing (siRNA) experiments were designed (see flow chart in Figure 5A). In preliminary studies, a dose-dependent increase of apoptotic signal (caspACE VAD) was detected after exposure to increasing NGF, resembling those of both AnnexinV and cleaved caspase3. Electroporation efficiency was estimated to be around 70% to 88% (counting GFP positive cells; Figure 5B). In accordance with the molecular data, flow cytometry analysis showed that a proportion of p75^NTR^ protein was still present in the culture system, as shown in Figure 5C-D. Confluent control-siRNA and p75^NTR^-siRNA electroporated cells were exposed to 100 ng/mL NGF or 10 ng/mL TGFβ1 and harvested after 3 days to evaluate and compare the apoptotic signals. As shown in Figure 5E, electroporation with 1.5 µg/mL p75^NTR^-siRNA was effective in reducing the apoptotic signal (35%-decrease in apoptosis) in 100 ng/mL NGF treated cells, as compared to 100 ng/mL NGF-treated control siRNA cells (referred as 100% apoptosis). Control-siRNA had no effect on apoptotic signal. This low but significant reduction of apoptotic signal might be explained by the presence of trkA^NGFR^. Therefore to verify this explanation, a double silencing of both trkA^NGFR^ and p75^NTR^ was performed next and electroporated cells were exposed to 100 ng/mL NGF or 10 ng/mL TGFβ1 (positive control). Even in the presence of both receptor silencings, apoptosis was not completely abolished in 100 ng/mL NGF treated cells (47–50%-decrease in apoptosis over untreated cells (100%)). The results demonstrate that p75^NTR^-siRNA is effective in reducing the apoptotic signal induced in NGF-treated myoFB, while trkA^NGFR^-silencing might make some contribution. A slight apoptotic signal was also detected in untreated myoFB and TGFβ1-treated ones.

## Discussion

The main finding of this study indicates that TGFβ1-induced conjunctival myoFB (herein referred as myoFBs) significantly increase their p75^NTR^ and undergo apoptosis upon acute and particularly chronic NGF treatment. Both chemical inhibition and single- (p75^NTR^) and double (trkA^NGFR^/p75^NTR^)-silencing approaches (siRNA) down-regulated the apoptotic signal, highlighting the contribution of NGF/p75^NTR^ in mediating myoFB apoptosis.

Tissue remodeling and fibrosis clearly compromise ocular surface structure and lead to ocular function decline [1], [2]. FB/myoFB, known to drive healing response, can be modulated at various levels. The injured ocular microenvironment as well as tears and aqueous humor contain several cytokines, growth factors and chemical mediators that greatly power the local response, contributing as profibrogenic mediators [2]. We previously reported the NGF ability to stimulate *in vitro* the induction of myoFB phenotype and matrix contraction, highlighting NGF contribution at both early and late stages of proper (physiological) resolution of tissue repair [13], [14], [18]–[21]. Focused studies on these FB demonstrated that all the profibrogenic NGF effects were partially mediated by modulation of TGFβ1 [13], [14], [18], in line with findings provided in other systems as well as in animal models [22], [23].

In order to investigate the NGF effect on myoFB, a cell culture model of TGFβ1-induced myoFB was reproduced and firstly characterized for NGF receptor pathway. TGFβ1 is a widely accepted chief inducer of FB differentiation, even if overlapping mechanisms and soluble factors might contribute massively to the development of myoFB phenotype in inflamed tissues, [17]. In this model, FB differentiate into myoFB upon TGFβ1 exposure, shifting their morphology from a typical flattened irregular shape (FB) to an elongated and spindle shaped appearance (myoFB), associated with a consistent αSMA expression [3]. These myoFB showed a significant increase of αSMA and p75^NTR^ expression, and preserved trkA^NGFR^ expression, in comparison to quiescent αSMA negative conjunctival FB expressing only trkA^NGFR^ and/or low levels of p75^NTR^ [11], [24]. To the best of our knowledge, this is the first evidence of TGFβ1 as an inducer of trkA^NGFR^/p75^NTR^ expression, while NGF induction of TGFβ1 has been reported for other cell types [13], [14]. p75^NTR^ expressing myoFB have been detected in fibrotic tissues, where myoFB differentiation is mainly mediated by TGFβ1 [18], [25].

Next, the NGF receptor pathway was investigated in stimulating and neutralizing experiments. Acute and massively chronic NGF exposure triggered a selective increase of p75^NTR^ expression while trkA^NGFR^ expression was slightly modulated. Both treatments shifted the trkA^NGFR^/p75^NTR^ rate toward p75^NTR^ expression, in a dose- and time-dependent manner, resulting in a decrease of the trkA^NGFR^/p75^NTR^ ratio. As previously reported, p75^NTR^ expression is a typical feature of healing myoFB [20], [24]. Indeed, NGF effect was associated with a significant decrease of αSMA and TGFβ1 profibrotic genes, the main markers of myoFB survival. An inverse correlation was detected between αSMA and p75^NTR^ gene expression. The higher trkA^NGFR^/p75^NTR^ ratio in untouched FB (data not shown), the slight decrease in induced myoFB and the significant decreased upon NGF exposure in favor of p75^NTR^ expression, suggest a possible NGF-p75^NTR^ involvement in myoFB survival.

As previously reported, conditions leading to a lower trkA^NGFR^/p75^NTR^ ratio are often associated with apoptosis [12], [15], [24]. An increase in the number of rounded/condensed cells with shrinkage of cytoplasm (monolayers) and several free-floating cells (supernatant) were observed after acute and chronic NGF treatments. As confirmed by *in situ* end labeling of oligonucleotyde fragments (TUNEL), a proportion of myoFB had fragmented DNA, an effect not observed when cells were exposed to TGFβ1 [26]. To discriminate early and late apoptotic cells from necrotic ones, a specific AnnexinV/PI analysis was carried out: early apoptotic cells were selected from live and dead cells by cell sorting of AnnexinV labeling and exclusion of vital dye. This AnnexinV/PI study extended the above mentioned results showing an increased number of late apoptotic cells upon chronic treatment. Since NGF effects were studied in myoFB cultured in medium supplemented with 1% FCS, mimicking the microenvironment rich in growth factors, a counteracting serum effect on NGF apoptosis should not be excluded. More interestingly, AnnexinV positivity was drastically reduced in cells treated in the presence of neutralizing anti-NGF antibodies or in cells pretreated with chemical receptor neutralizers, suggesting a task for NGF and its receptors at the resolution of the repair process.

Arising from quiescent FB and transiently expressed during wound-healing, myoFB play a crucial role in the late phases of the repair process being responsible for the ECM deposition and contraction [2]–[4], [17]. MyoFB disappearance has been documented during remodeling stages or in fibrotic tissues, suggesting that a gene-directed cellular self-destruction (apoptosis) is necessary for resolution of fibrosis [27]. Unwanted persistence of myoFB could be one of the causes for excessive scarring and fibrosis [28], [29]. MyoFB resistance to apoptosis seems to be strongly regulated by cytokines and growth factors, which might influence the microenvironment [5]. TGFβ1 is unequivocally recognized as inducing survival of myoFB both *in vitro* and *in vivo*, even though other growth factors and cytokines might contribute significantly [5], [26]. The observation that apoptosis paralleled TGFβ1 and αSMA down-regulations might suggest a possible NGF modulation of myoFB survival directly by inducing apoptosis or indirectly by decreasing TGFβ1 levels [29]. In line with this observation, TGFβ1 is also a survival factor for myoFB and is always increased in fibrotic tissues [5], [17], [26]. In the context of *in vivo* fibrotic-tissue system, we might hypothesize that NGF might trigger “selective” apoptosis, after being released by (primed)-FB/myoFB and other similar cell types populating the wounded area. From our study, TGFβ1 continued to stimulate NGF productions (data not shown). This hypothesis is in line with previous studies indicating that fetal FB express high amounts of NGF and scarring phenomena not present during the fetal period and that NGF topical application allows proper healing and does not trigger fibrosis in injured tissues [30], [9], [19], [23].

In our study, the cells showing DNA fragmentation were also recognized as p75^NTR^ bearing cells, as detected by sorting analysis. NGF exposure resulted in increasing cleavage of caspase-3 significantly expressed in p75^NTR^ bearing myoFB. Even though in this cell culture model, the apoptotic pathway induced by NGF appears associated with activation of the effector caspase-3, the intracellular death-pathway induced by p75^NTR^ activation is unknown.

According to the literature, trkA^NGFR^ mainly drives proliferation, differentiation and survival while p75^NTR^ mainly triggers differentiation and apoptosis [12], [15]. Specific single/double neutralizing experiments showed the contribution of both receptors in PS outer-expression and caspase-activation. As an explanation, both receptors are present on the surface either in homo and/or hetero dimeric state and depending on the magnitude/specificity of the binding, both receptors can drive a response [15], [16].

Therefore to provide additional information, apoptosis was also investigated in NGF-treated myoFB expressing low trkA^NGFR^/p75^NTR^ ratio (100 ng/mL) by using small inhibitory RNAs (siRNA) directed against p75^NTR^ [31]. Specific silencing of p75^NTR^ significantly reduced the percentage of apoptosis after 100 ng/mL NGF exposure, as observed in comparison to control siRNA myoFB. However, a complete reduction of apoptotic signal was not detected. According to the literature, p75^NTR^ seems to allow apoptosis in receptive cells either directly (ceramide and caspase activation) or indirectly by the modulation of trkA^NGFR^ trafficking/signaling [15], [16], [32]. Indeed, trkA^NGFR^-mediated apoptosis has been also documented, ascribed to a novel Ras and/or Raf signaling pathway [33]. Since trkA^NGFR^ is primarily, but not exclusively, a proliferating and survival receptor, cells were subjected to double knocking-down experiments. Still in the presence of double trkA^NGFR^/p75^NTR^ silencing, apoptosis was as low as those of single p75^NTR^ silencing, but not completely abolished. In our studies of silencing, both single and double trkA^NGFR^/p75^NTR^ target gene amplification confirmed only a partial decrease in mRNA expression. Besides fitting transfection efficiency, some proteins still persist in the system even after 72 hrs from gene knocking down: a change in the RNA level for a particular gene product does not directly correlate with a change in the amount of protein in the cell. This explanation could fit our results to explain the incomplete p75^NTR^ and trkA^NGFR^/p75^NTR^ protein silencing. An additional explanation is that in this system, NGF might not be the only factor responsible for myoFB induced apoptosis. Taken together, the explanations for a 37%-decrease in apoptosis during single- and 47%-decrease in apoptosis during double-silencing RNA experiments might be due to an incomplete silencing of technique, an intracellular p75^NTR^ retaining, an enhanced trkA^NGFR^ and/or p75^NTR^ signaling, or other indirect factors induced by NGF signaling [8], [16], [23], [32], [33]. Several data on experimental models showed the selective expression of p75^NTR^ in myoFB within fibrotic tissues and the p75^NTR^ contribution to myoFB differentiation and cell apoptosis [25], [34]–[37]. At least in part, these *in vitro* results might provide an explanation for the observation that NGF does not induce a massive apoptotic effect on *fibrotic tissues*.

Taken together, our in vitro model provides data on NGF as an inducer of apoptosis by shifting the trkA^NGFR^/p75^NTR^ ratio in favor of p75^NTR^ and inducing up-regulation of terminal caspase-3, opening a more fruitful possibility for counteracting myoFB resistance to apoptosis. Indeed, the observed NGF-mediated TGFβ1 deregulation is in line with the apoptotic effect and with the inhibition of TGFβ1-mediated myoFB endurance. In line, our data might explain the observation of the successful corneal healing upon NGF topical application [9], [19]. It still remains to be elucidated whether during chronic inflammatory responses characterized by increased p75^NTR^ and NGF levels, NGF might improve apoptosis of myoFB in the presence of pro- and/or anti-apoptotic factors (TGFβ1, IL1β, PDGF, FGF, IL4, IL6) [38]–[39]. Cumulatively, these findings would suggest new therapeutic strategies to offset overt fibrosis by modulating myoFB survival in fibrotic tissues.

## Materials and Methods

### Reagents

Purified 2.5S-NGF (GradeI) and purified human TGFβ1 were provided by Alomone Labs Ltd. (Jerusalem, Israel) and PeproTech EC Ltd (London, UK) respectively; neutralizing anti-pan TGFβ antibodies and neutralizing anti-NGF antibodies (500 ng/mL) were from R&D Systems, Minneapolis, MN; neutralizing anti-trkA^NGFR^ (100 ng/mL) and anti-p75^NTR^ (100 ng/mL) were from Calbiochem Novabiochem Corp. (San Diego, USA). Conjugated specie-specific secondary antibodies were from Jackson ImmunoResearch Europe Ltd (Suffolk, UK). Other analytical grade chemicals and solvents were from ICN (Costa Mesa, CA), SERVA (Weidelberg, Germany) and Sigma Chemicals (St Louis, Mo), unless otherwise indicated. All tissue culture reagents were from Lonza (Basel, Switzerland) and sterile tissue culture plastic-ware were from NUNC (Roskilde, Denmark). Grade reagent and Ultrapure/RNAse free water was provided by Direct-Q 5 Apparatus (Millipore, Vimodrone, Milan, Italy).

### The *myoFB* Model

The research protocol followed the tenets of the Declaration of Helsinki and was approved by the Intramural Committee of Hadassah University Hospital. Human primary cultures of conjunctival FB (passages 3–7) were obtained from Innoprot (Bizkaia, Spain) and used for these studies. Cell cultures were expanded (24 hrs doubling time) in DMEM supplemented with 10% heat-inactivated Fetal Calf Serum (FCS), 2 mM-glutamine, 100 U/ml pen-streptomycin, under standard culturing conditions and re-plated after trypsin-EDTA harvesting. To obtain *in vitro* myoFB, preliminary titration experiments were carried out with increasing TGFβ1 concentrations (0.1, 1, 2, 5, 10 and 20 ng/mL) in serum free conditions [40]. Both molecular and biochemical analysis of αSMA and myoFB/FB rate expression identified 2 ng/mL TGFβ1 over 3d for almost 70–80% myoFB transdifferentiation. In some experiments, enriched myoFB (99% myoFB) were obtained after selection (EasySep®Magnet, Stem Cell Technology, Voden Medical Instruments, Milan, Italy) with anti-αSMA antibodies conjugated to magnetic biodegradable beads (Miltenyi Biotec, GmbH, Germany), according to the manufacturer’s procedure.

### Experimental Procedure

TGFβ1-induced myoFB (herein termed myoFB and defined as αSMA-expressing myoFB) [17], [40], were re-plated at high density in the absence of FCS to retain αSMA phenotype. At confluence, monolayers were exposed to increasing doses of NGF in the presence of 1% FCS (to guarantee synchronization and reduction of autophosphorylation) over the specified time-points. Acute and chronic NGF exposures were performed as follows: acute exposure was a single stimulus and chronic was three stimuli repeated every two days (NGF replaced in fresh medium). Acute and chronic samplings were performed at 5, 12, 24 and 48 hrs from last stimulus, depending on the evaluation. For neutralizing experiments, monolayers were exposed to chemical inhibitors or specific antibodies for 30 min prior to the addition of NGF. The starting-time point (control) indicates untreated myoFB re-plated and harvested in parallel with NGF treated myoFB. Cells were trypsin harvested and washed in Hank’s Balanced Salt Solution (HBSS without Ca^2+^/Mg^2+^), before biochemical or molecular evaluations. For most of the experiments, TGFβ1 exposure was carried out in parallel, according to the survival effects of TGFβ1 on myoFB [26].

### MTS Assay and Cell-surface ELISA

Cells were seeded on 96-well plates and then treated with increasing NGF doses and times depending on the experiment. Cell viability was determined by measuring mitochondrial reduction of the MTS dye reagent into a soluble formazan product, according to the manufacturer’s instructions (Promega, Madison, WI). Cell surface ELISA was carried out for quantifying αSMA expression, as previously reported in detail [13], [14], [18]. Absorbance measurements (490 nm) were recorded using a Sunrise spectrophotometer (Tecan Systems, Inc., San Jose, CA).

### Flow Cytometric Analysis

Single cells (10^6^cells/well) were processed for membrane/cytoplasm staining (PE-trkA^NGFR^/FC-p75^NTR^ diluted 1/50; Santa Cruz Biotec, Santa Cruz, CA), according to a standardized protocol including mild postfixation (0.3% ρ-formaldehyde (PFA)), brief methanol permeabilization, blocking (5% FCS) treatments and staining in PBS containing 5% FCS, 1 mM EDTA and 0.05% NaN_3_ (FACS buffer). Cells were incubated with mouse anti-human αSMA antibodies (sc-130616) followed by species-specific APC-conjugated anti-Mouse IgG antibodies; or probed with a PE-conjugated anti-human trkA^NGFR^ antibodies (sc-118) and FC-conjugated anti-human p75^NTR^ antibodies (sc-81612) mixture (Santa Cruz Biotech., Santa Cruz, CA). Cleaved caspase-3 antibodies (Asp.175 #9661; Cell Signaling Technology Inc., Danvers, MA) were labeled with PerCP specie-specific antibodies. Cells were evaluated using a digital based flow cytometry station (MACSQuant Analyzer, Miltenyi). Hyperlog (hLog) signals were analysed from 5000 gated cells/sample. Instrument calibration was checked weekly by use of Microbeads (Miltenyi) and individual compensation settings for each separate reagent combination (tube-specific compensations) were performed by use of antibody-capture beads (CompBeads, Becton Dickinson, San Jose, CA). Instrument settings were performed using control (isotype-matched antibodies from eBiosciences, San Diego, CA), single and double fluorescent samples, run in parallel for each set of experiments. MFI of hLog distribution was calculated, non-specific signal for each sample was subtracted from the specific one, and results were expressed as increments relative to the controls, calculated as follows: ΔMFI = (specific MFI - non-specific MFI)/non-specific MFI. A MFI ratio>1 represents significant expression. Histogram or density plots were arranged using the MACSQuant Digital software. MFI data are expressed as means±SD.

### Confocal Laser Microscopy

Cells on round coverslips (2x10^5^) were washed in 10 mM Phosphate-Buffered Saline (PBS, pH7.5) at the stated time-points, fixed in 3.7% buffered PFA, quenched in 50 mM NH_4_Cl to rule out autofluorescence and blocked/permeabilized in 3% BSA and 0.03% Triton X-100 (TX) in PBS. Monolayers were probed with: rabbit anti- trkA^NGFR^ antibodies and goat anti- p75^NTR^ antibodies (4 µg/mL, both from R&D); mouse anti-αSMA (2 µg/mL; Santa Cruz) and active caspase3 (Cell signaling). Specific binding of the primary antibody was detected using Cy2 or APC-conjugated secondary antibodies (1/500). Nuclear counter-staining was performed with Propidium Iodide (25 µg/mL) or with TOTO-3 (1 mM) in PBS containing RNase (20 µg/mL) and coverslips were mounted in hand-made anti-fade medium. Irrelevant isotype-matched IgG antibodies (Vector) were incubated in parallel and used as internal controls for the channel series acquisitions and related background subtraction. Monolayers were examined and images were acquired using an inverted E2000-U microscope equipped with the C1 software (x20/0.45NA; x40/0.60NA; x60/1.4 oil; Nikon, Tokyo, Japan). TIFF-converted pictures were assembled by Adobe Photoshop 7.0 (Adobe Systems Inc., San Jose, CA).

### Relative Real-Time PCR

Total RNA was extracted from cells (2×10^5^) treated and harvested at 5 or 24 hrs from last stimulation, using the EuroGold TRIfast™ kit (Euroclone, Milan, Italy) and diluted in RNase free water (Millipore). The concentration and purity (260/280 nm and 260/230 nm) of total RNA were determined by using a spectrophotometer (NanoDrop® ND-1000, Wilmington, DE, USA), while 28S/18S ratios were recorded after agarose gel separation. When required, a DNAse treatment was allowed to guarantee absence of contaminating DNA (Turbo DNA free kit; Ambion, Milan, Italy). Only RNAs showing RNA/DNA rate >1.8 were used for analysis. Real time PCR was performed in a two step manner: cDNA synthesis and amplification were carried out in a One Personal thermocycler (PeqLab, EuroClone) and in an Opticon2 system (MJ Research, Watertown, MA), respectively. Oligo dT_21_-primer was used to generate cDNA from 1 µg total RNA, according to the manufacturer’s instructions (Improm kit; Promega). SYBRGreen HotStart AmpliGold Taq polymerase (Applied Biosystems, Foster City, CA) was used for specific amplification of 3 µL cDNA in a final volume of 20 µL in 96-well plate including internal controls, at stated amplification settings (see Table 1). In preliminary studies, electrophoresed amplicons were purified with a gel extraction kit (Wizard SV gel system, Promega) and sequenced on a DNA sequencing system (ABI PRISM 3700; Applied Biosystems). Each RT-PCR reaction contained equivalent amounts of total RNA (normalized samples). Target gene expression ratio was calculated from all single Cts (both target and referring genes in duplicate per sample) run in the Relative Expression Software Tool REST-384^©^ ver.2 [41]. Data are expression ratios in 2log scale relative to treated *vs.* untreated myoFB and untreated myoFB *vs.* untreated FB. As negative controls, amplification was performed in the presence of total RNA (exclusion of genomic contamination) or on cDNAs obtained from reverse transcription tubes in the absence of enzyme (exclusion of unspecific amplification).

**Table 1:** Primer schedule.

aGene(Access no)	Sequence	Amplicon (bps)	Annealing conditions^c^
**GAPDH**(BC013310)	F: 5′-GAA GGG GTC ATT GAT GGC AAC-3′R: 5′-GGG AAG GTG AAG GTC GGA GTC -3′	100 bps	53°C, 30 sec
**TGFβ1**(BC017288)	F: 5′-TCC TGG CGA TAC CTC AGC AA-3′R: 5′-GCC CTC AAT TTC CCC TCC AC-3′	110 bps	53°C, 30 sec
**α-SMA**(BC017554)	F: 5′-GAA GGA GAT CAC GGC CCT A-3′R: 5′- ACA TCT GCT GGA AGG TGG AC-3′	125 bps	60°C, 25 sec
**NGF**(V01511)	F: 5′- CTG GCC ACA CTG AGG TGC AT-3′R: 5′- TCC TGC AGG GAC ATT GCT CTC-3′	120 bps	53°C, 30 sec
**trkA^NGFR^** (M23102)	F: 5′-CAT CGT GAA GAG TGG TCT CCG-3′R: 5′-GAG AGA GAC TCC AGA GCG TTG AA-3′	103 bps	57°C, 25 sec
**p75^NTR^**(AF187064)	F: 5′-CCTACGGCTACTACCAGGATGAG-3′R: 5′-TGGCCTCGTCGGAATACG-3′	147 bps	57°C, 25 sec
b **siRNA** **(** ***Access no*** **)**	**Sequence:**		
**si-p75^NTR^**(AF187064)	S: 5′- CCGUUUGCUGUGAACCCUAUGUUAU -3′AS: 5′- AUAACAUAGGGUUCACAGCAAACGG -3′		
**si- trkA^NGFR^** (M23102)	S: 5′- CCAUCGUGAAGAGUGGUCUCCGUUU -3′AS: 5′- AAACGGAGACCACUCUUCACGAUG G-3′		

A summary of primer names, for/rev primer sequences (5′ to 3′), PCR product size (amplicons in bps), annealing conditions and Genebank accession numbers of each gene investigated. F: forward primer; R: reverse primer.

a PCR primers were designed by Primer3 software (genome.wi.mit.edu/cgi-bin/primer/primer3_www.cgi) and synthesized by MWG (mwg.com/; Ebersberg, Germany).

b The RNAi were designed by Invitrogen website (rnaidesigner.invitrogen.com/).

c Amplification parameters were as follows: 37 cycles of 30 s/94°C, 25s/specific Ta, 30 s/72°C, preceded by 15 m/95°C hot start polymerase activation and followed by fluorescence monitoring during linear transition from 55–90°C, 0.01°C for 0.3 sec and further 5 m/72°C incubation.

### Apoptosis Detection

Conditioned media were gently removed and centrifuged to collect death cells, and adherent cells were briefly trypsin harvested and processed as reported in detail.

#### Hoechst/DAPI staining

Cells (2x10^5^) were treated with NGF for 5 hrs and stained with 1 µg/mL Hoechst 33342 (HO342) for 5 min at 37°C or 1 µg/mL DAPI for 15 min at 37°C (both from Invitrogen-Molecular Probes, Milan, Italy) to identify cells with membrane shrinkage, chromatin condensation and clumping or enlarged cells. Monolayers were photographed with a microscope equipped with epifluorescence to detect Hoechst/DAPI signals and software for image acquisition (Nikon, Tokyo, Japan). TIFF-converted pictures were assembled by Adobe Photoshop 7.0.

#### TUNEL staining

Adherent cells (2×10^5^) were stained with the biotinyled dATP nick end labeling procedure (TUNEL), which mark DNA fragmentation. Briefly, fixed (3.7% PFA) and permeabilized (0.1% TX) cells were incubated with exogenous rTdT enzyme and biotin 14-dATP (37°C/3 hrs; Invitrogen), for repair of 3′-hydroxyl DNA ends. Positive controls were carried out in parallel, with a DNase I pre-incubation of monolayers (2 U/mL; 37°C/30 min). Negative controls were monolayers that were incubated with buffer lacking rTdT enzyme. The apoptotic nuclei were labeled according to the ABC technique (Vector Laboratories, Burlingame, CA) using DAB solution (Dako Corp., Carpinteria, CA) as substrate. TUNEL positive cells were scored by two independent observers (blind fashion), and five optic fields/coverslip for at least 10 coverslips were counted for each culture condition (x20). The percentage of apoptosis was calculated as (number of apoptotic cells/number of total cells)×100. For double staining with p75^NTR^ and specific FC-secondary antibodies, TUNEL reaction was performed according to the TRITC-Avidin procedure (Vector). Stained cells were imaged as reported in the confocal laser microscopy section.

#### FACS analysis of apoptosis

Single harvested cells (10^6^) were equilibrated in Hepes buffer containing 2 mM CaCl_2_, and subjected to immunofluorescence as follows. To detect externalized PS, cells were probed with FC-AnnexinV-Apoptosis detection kit (Miltenyi), according to the manufacturer’s directions with the exception that cells were post treated with RNAse A and finally fixed in 3% PFA [42], [43]. Controls were prepared according to the recommendation of the supplier. Single harvested cells were double-stained with cleaved caspase3 and APC conjugated anti-Rabbit secondary antibodies and FC-conjugated anti-p75^NTR^ antibody in FACS buffer. Logical gate (back-gating on FL2-FC) combining p75^NTR^ positive cells and their scatter properties were used for select cleaved caspase-3 positive cell population. CaspACE-VAD and cleaved caspace3 quantifications were carried out according to the manufacturer’s instructions (Promega). Acquisitions and analyses were carried out as above described in detail in the FACS session.

### Small Interference RNA (siRNA)

Specific Stealth™ siRNA molecules were chemically synthesized, as desalted, 25mer duplex oligonucleotides (Table 1) (rnaidesigner.invitrogen.com/). The Nucleofection was performed in an electroporation machine (Amaxa GmbH; Instrumentation Laboratory, Milan, Italy). To set up the technique, preliminary experiments were carried out according to the optimized protocols of the manufacturer’s instructions (Amaxa’s silencing kit). Three different silencing concentrations (0.5, 1 and 1.5 µg/mL) were tested and 5–72 hrs after plating, electroporated cells were screened for the highest % of efficiency (80–90%) in knock-down gene expression, by specific mRNA (PCR) and protein (FACS) analysis. For serial specific experiments (1.5 µ/mL siRNA): single-cells (10^6^) were re-suspended in 100 µL specific buffer (under serum-free conditions), mixed with GFP marker and siRNA sequences [single (p75^NTR^-siRNA) or both (trkA^NGFR^/p75^NTR^-siRNA) or non-specific (control siRNA)], quickly nucleofected, harvested in 1 mL pre-warmed culturing medium and seeded in 6 well plates or T21cm2 plates or 4-chamber slides (Nunc) depending on analysis. Transfected cells were cultured and fed daily with fresh medium until they were stimulated and assayed (flow-chart in Figure 5A).

### Statistical Analysis

All the experiments were done independently at least three times, each experiment being carried out in duplicate or triplicate. Data (means±SD) were exported to Excel datasheet and presented as graphs (Excel Microsoft Corp., Redmond, WA; Microsoft.com/excel). Statistical analyses were performed by using the StatView II package for PC (Abacus Concepts. Inc., Berkeley, CA). All data followed a normal distribution and parametric ANOVA followed by Tukey-Kramer post hoc was employed to analyze data. The Spearman’s rank correlation coefficient (Rho) was calculated to identify specific target relationships. A confidence of 95% was presumed to reflect significant difference between group mean values, and therefore a p-value of <.05 was taken as the limit of significance. A specific REST/ANOVA coupled analysis was carried out for PCR experiments.
